# *PROSPERO*: online prediction of crystallographic success from experimental results and sequence

**DOI:** 10.1107/S002188981201775X

**Published:** 2012-05-16

**Authors:** Frank H. Zucker, Hae Young Kim, Ethan A. Merritt

**Affiliations:** a Biomolecular Structure Center, Department of Biochemistry, University of Washington, Seattle, WA 98195-7742, USA

**Keywords:** protein crystallography, protein characterization, *PROSPERO*, computer programs

## Abstract

The growth of diffracting crystals from purified proteins is often a major bottleneck in determining structures of biological and medical interest. The *PROSPERO* web server, http://skuld.bmsc.washington.edu/prospero, is intended both to provide a means of organizing the potentially large numbers of experimental characterizations measured from such proteins, and to provide useful guidance for structural biologists who have succeeded in purifying their target protein but have reached an impasse in the difficult and poorly understood process of turning purified protein into well diffracting crystals. These researchers need to decide which of many possible rescue options are worth pursuing, given finite resources. This choice is even more crucial when attempting to solve high-priority but relatively difficult structures of eukaryotic proteins. The site currently uses the *HyGX1* predictor, which was trained and validated on protein samples from pathogenic protozoa (eukaryotes) using results from six types of experiment. *PROSPERO* allows users to store, analyze and display multiple results for each sample, to group samples into projects, and to share results and predictions with collaborators.

## Abstract 2

The *PROSPERO* server analyzes sequence and experimental protein characterization results, then uses that analysis to predict crystallization outcome and suggest priorities for futher work on difficult targets. The server allows users to upload data from six types of experiment, to organize those data by sample and project, and to share those data with collaborators.

## 1. Introduction

Three-dimensional structures of proteins provide valuable information for scientific and medical research. Structures of proteins from humans and from human pathogens, both eukaryotic and bacterial, are especially useful for structure-based drug design. However, eukaryotic proteins expressed in the commonly used bacterial heterologous expression systems are generally more difficult to express solubly and to crystallize than proteins from other kingdoms. For example, the success rate for eukaryotic targets from several large structural genomics centers tracked in TargetDB (Chen *et al.*, 2004 ▶), *i.e.* the fraction of cloned targets that yield crystal structures, is only 15% of the success rate for archaeal and bacterial targets. This lower success rate is consistent with outcome summaries from other individual projects with eukaryotic targets (Mehlin *et al.*, 2006 ▶). Thus it is particularly important to properly prioritize crystallization efforts for eukaryotic proteins.

Researchers need to prioritize their follow-up efforts for targets that yield no crystals on initial screening, which constitute at least two-thirds of all targets tracked in TargetDB. Possible follow-up approaches include (i) substitution of homologous proteins from related species; (ii) mutations to reduce surface entropy or improve crystal packing; (iii) sequence truncation; (iv) alternative choices for expression vector, affinity tags and solubility tags; and (v) alternative expression hosts, expression conditions and purification protocols. The combinatorial space of options created by mixing and matching these alternatives is very large. A predictive tool to aid in prioritizing which variations are most likely to succeed would clearly be desirable.

Several previous tools have been developed to predict the likelihood of obtaining diffracting crystals of purified proteins based on sequence information only, *e.g.* *XtalPred* (Slabinski *et al.*, 2007 ▶) and *Pxs* (Price *et al.*, 2009 ▶). These tools were optimized using data mainly from prokaryotic and archaeal proteins. Perhaps as a result, they do not predict eukaryotic crystallization well (Price *et al.*, 2009 ▶; Zucker *et al.*, 2010 ▶). Furthermore, it is well established that small changes such as mutation of a single residue or short truncations of the N or C terminus may dramatically change the physical or crystallization properties of a protein (Cooper *et al.*, 2007 ▶; Klock *et al.*, 2007 ▶; Gräslund *et al.*, 2008 ▶). Unfortunately, sequence-based predictors are insensitive to such minor changes.

We have developed a predictor, *HyGX1*, that uses a combination of sequence and experimental results from proteins of eukaryotic origin (Zucker *et al.*, 2010 ▶). *HyXG1* is a recursive regression partition tree trained on sequence and experimental results from 77 samples from a structural genomics project targeting pathogenic protozoa. The predictor was validated on a distinct set of 30 protozoan proteins. No membrane proteins or multi-protein complexes were included in either the training or validation sets. The current predictor uses values from four types of physical experiment and two values from sequence analysis (molecular weight and disorder) to split samples into categories with different outcomes. The experimental values are derived from differential scanning fluorimetry (DSF), expression yield, size-exclusion chromatography (SEC) and dynamic light scattering (DLS). Outcomes are measured by a diffraction score that runs from 0 (no crystals) to 6 (crystals diffract to 2.0 Å or better). These predictions provide some guidance for researchers faced with difficult targets and limited resources.

Here we describe *PROSPERO*, a web interface to the *HyGX1* predictor. The *PROSPERO* web site allows users to upload sequence and experimental results, and returns estimates of the likely crystallization outcome along with suggestions for prioritizing next steps in the absence of initial crystal hits. The site also provides storage, sharing, analysis and display of experimental data, independent of its use for prediction. Programs for fitting SEC and DSF curves can be run on the server or downloaded and run locally. Data from several experimental types can be displayed in a standard format, allowing researchers to rapidly compare sample quality and make their own judgments.

## 2. The *PROSPERO* web server

The *PROSPERO* web server provides tools to input, track, share and analyze physical characterizations of protein targets, and uses these experimental results to estimate the likely outcome of crystallization and make suggestions for prioritization of further crystallographic efforts. A user manual is accessible *via* a ‘Documentation’ tab on the menu bar at the top of each page, with context-dependent help links to specific sections.

### 2.1. User access and data organization

Users can upload data and use the predictor anonymously. A free, minimal registration process gives users easier subsequent access to those private data, and allows them to create a password-protected account for shared data access.

**Figure 1:**
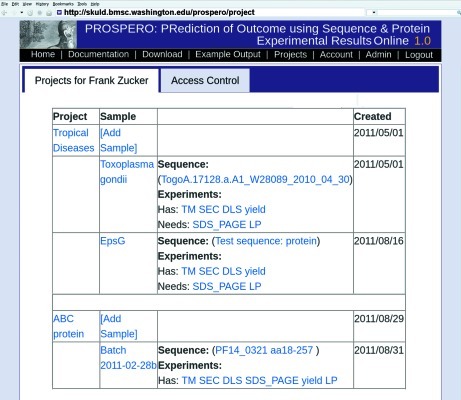
*PROSPERO* data organization. The user’s projects tab on the ‘all projects’ page. Users can have several projects, each comprising many samples with sequence and experimental results. Not shown: each sample can have one or more associated sequences and possibly multiple results from each type of experiment.

Samples within an account can be further subdivided into projects, *e.g.* all variants of one target, or sets of homologous genes. Users can select from a displayed table of their existing projects and samples, or add a new project or a new sample within a project (Fig. 1 ▶). For each project, sample and sequence, users can supply both a name (a short identifier to be used in lists and navigation tools) and a longer description (displayed when room is available).

Users can select items within the table for more detailed views. At each level of detail, the path down to that level is shown in outline form near the top, with links allowing the user to navigate back up to higher levels. Views available to the user include the following:

(*a*) All projects. The table shown in Fig. 1 ▶ has links to each project, sample, sequence and experimental type. It also indicates data status, *i.e.* which types of data have or have not yet been entered for each sample. From this ‘all projects’ page, users can add a project or select an existing project to view the ‘project’ page.

(*b*) Project. This page has the project description and a table with names, descriptions and data status of each sample in that project. From either the ‘project’ or the ‘all projects’ page, users can add a sample or select an existing sample to view the ‘sample’ page.

(*c*) Sample. The ‘sample’ page shows summary values for sequence and for each type of experiment and graphically indicates data status with colored bars. From this ‘sample’ page, the user can add sequence and experimental results and can submit the currently selected data for prediction of crystallization outcome.

(*d*) Result list. From any of the above pages, users can select sequence or experimental type to view the list of existing results and analysis for one type of data. This list of results includes summary values and a graphic thumbnail for each experiment, where available. From this page, users can add results, select one result to view the detailed analysis or toggle the selection of results for use in the predictor.

(*e*) Experiment. Each type of data has its own page displaying summary values and, where available, graphic representations of the experimental result and analysis.

### 2.2. Data input and intermediate analysis

In addition to the experimental data used by the current predictor (DSF, SEC, DLS, screening or large-scale soluble expression yield), the *PROSPERO* web server also accepts SDS gel images for protein purity verification and results from limited proteolysis. Perl input modules for DSF and SEC parse machine-specific data and metadata files into a standardized XML format used by *PROSPERO* for initial display during upload and for transferring data to the server’s database. These modules can also carry out analysis such as curve fitting to derive values useful to researchers and to the predictor. Users can either upload their raw experimental files directly for conversion to standardized format by the server itself, or download the input modules for standalone analysis or modification of input parsing, followed by upload of the resulting XML.

(*a*) Sequence-based data. Sequences can be pasted or uploaded in single-character notation. For single-protein samples, disorder is predicted using *DisEMBL* (Linding *et al.*, 2003 ▶). Average hydropathy is calculated using values from Kyte & Doolittle (1982 ▶). Sequences of prokaryotic proteins are also sent for external analysis to the *Pxs* (Price *et al.*, 2009 ▶) and *XtalPred* (Slabinski *et al.*, 2007 ▶) predictors. The current *HyGX1* predictor makes use of the molecular weight (MW) of the monomer estimated from the sequence and the longest contiguous stretch of disordered residues (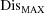) predicted by *DisEMBL*.

**Figure 2:**
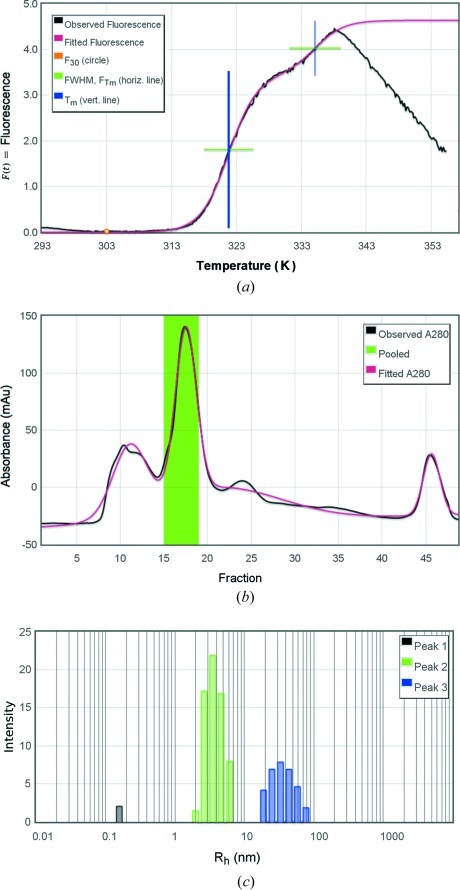
Graphical display of intermediate data analysis. (*a*) Differential scanning fluorimetry (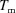) curve showing fluorescence intensity *versus* temperature; vertical bars are at transition midpoints (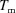) with a thicker bar for the major transition; horizontal bars are at 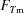 spanning the transition width (FWHM of the slope d*F*/d*T*). (*b*) Size-exclusion chromatography, absorbance in m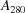 *versus* fraction, with superimposed models fitting multiple Gaussian peaks. (*c*) Dynamic light scattering histogram, plotted as intensity *versus* hydrodynamic radius.

Sequence data can be stored for multi-protein complexes or for samples containing DNA or RNA, but the current predictor was trained on single proteins and is not reliable for other types of sample. For protein complexes, the maximum molecular weight and maximum stretch of disorder will be used.

(*b*) Differential scanning fluorimetry (DSF). The input module tm_calc.pl takes comma-separated value (c.s.v.) files exported from Opticon Monitor RT-PCR machines using OM III software (Bio-Rad, Hercules, CA, USA), or from the Structural Genomic Consortium’s DSF analysis conversion tools (Niesen *et al.*, 2007 ▶). Other formats with temperature in one column and fluorescent intensities in following columns delimited by spaces or common punctuation marks can also be read. During upload, the input module converts all data in the input file to XML without curve fitting. The user chooses which curves should be analyzed by selecting from a list of well labels (*e.g.* from a 96-well plate of samples) as supplied in the input file, or selecting from thumbnails of the curves, or selecting the position within a schematic 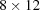 grid representing a 96-well plate. The input module is then run a second time using *gnuplot* (http://www.gnuplot.info/) for iterative Levenberg–Marquardt fitting of one or more Boltzmann transitions to the data up to the intensity maximum after the highest temperature transition (Fig. 2 ▶ *a*). For each transition, the midpoint (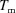), maximum slope (d*F*/d*T*), transition width (FWHM of d*F*/d*T*) and fluorescence change (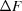) are reported numerically and graphically. On upload, the steepest transition is taken as the major one; users can alter this choice after curve fitting, *e.g.* if a different transition has a larger 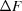. Fluorescence intensity at 303 K (*i.e.* at 30°C, 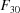) and at the midpoint of the major transition (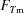) are used to calculate 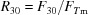, which is used by the current predictor.

(*c*) Size-exclusion chromatography (SEC). The input module sec_calc.pl takes text files of absorbance curves (curve.asc files) and run log documentation (doc.asc) extracted from AKTA *PrimeView Evaluation* software (GE Life Sciences, Piscataway, NJ, USA) and uses the log metadata such as flow rate, collection start time and fraction size to translate from time or volume units to fractions. During upload, users can enter or graphically select the range of fractions pooled to make the sample. After the pool range is entered, the input module is used again to make initial estimates of peak height and center for iterative fitting of Gaussian peaks using *gnuplot* (Zucker *et al.*, 2010 ▶) (Fig. 2 ▶ *b*). Supplying the pooled range allows calculation of percent purity of the pooled fractions (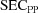), which will likely be of interest to the researcher. The fitted curve and each of its component Gaussians are displayed graphically. The key value for the current predictor is 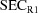, the residual after fitting a single Gaussian peak to the absorbance curve.

(*d*) Dynamic light scattering (DLS). Text files exported from DynaPro .exp files using *Dynamics* software (Wyatt Technology, Santa Barbara, CA, USA) are processed to extract and display a table of mean hydrodynamic radius, polydispersity, percent polydispersity, percent intensity and percent mass for each peak. The intensity *versus* radius histogram is also extracted and displayed (Fig. 2 ▶ *c*). The molecular weight of each peak, 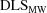 is calculated from the hydrodynamic radius and displayed in the same table.

The major peak is initially chosen as the highest intensity peak with hydrodynamic radius in the range 2–10 nm. This choice can be changed by the user after upload. For the major peak, 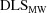 is used to calculate 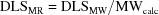, where 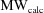 is the weight of the monomer estimated from the sequence. *PROSPERO* also calculates 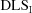, the ratio of the intensity of the major peak to the total intensity of all peaks excluding particles smaller than the major peak. The server also reports the DLS score (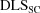), which categorizes DLS results according to the number of peaks and percent polydispersity. In the current predictor, 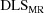 is the key value from DLS experiments.

(*e*) Yield. The yield of expressed protein from high-throughput screening can be entered as a visual score based on the examples provided in the input form, ranging from ‘none’ to ‘extremely high’ (equivalent to approximately 100 mg per liter of culture). The gel image can be uploaded for sharing and later reference. Large-scale expression yield can also be entered as mass of purified protein and volume of cell culture. The predictor uses the visual gel score when provided or the calculated number of milligrams per liter of culture otherwise.

(*f*) SDS–PAGE. A gel image and a visual gel score can be loaded and shared on *PROSPERO* to allow confirmation or reevaluation of purity. The data set of crystallization outcomes used to construct and train the current predictor contained insufficient data on outcomes of proteins with poor gels, as these had been dropped from consideration before conducting crystallization trials. Therefore the current predictor does not use this purity score.

(*g*) Limited proteolysis (Lp). Gel images and visual stability measures can be loaded. Stability is scored by estimating the change in MW and intensity of the major band before and after digestion for 1 or 24 h. The server uses these estimates to calculate a stability score from 1 (unstable) to 5 (extremely stable) for one or more proteases (Zucker *et al.*, 2010 ▶), and takes the average of those scores for all proteases. Among our samples, we found that proteins that are extremely stable in a variety of proteases are very likely to produce well diffracting crystals (Zucker *et al.*, 2010 ▶); however, there were too few such proteins to make this value statistically significant, and it is not used in the current predictor.

#### 2.2.1. Multiple results

For each type of experiment, users can enter multiple results and can view a list of those results. Users can then select which results are to be used in the predictor. The list displays the predictive values and other summary data for each result and displays graphic thumbnails for each selected result if available. If the user has selected more than one result for an experimental type, the server uses the mean of the scores from the selected results in the sample summary page and in predicting outcome.

#### 2.2.2. Manual data entry

The intermediate analysis of raw experimental data by the server can be bypassed by manually entering summary values from the experiment. This allows running of the prediction algorithm but not the graphical display of the results. For example, if the user has 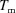, 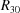 and 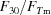 from DSF but does not have the full fluorescence *versus* temperature curve, those values can be entered into a web form rather than uploading a c.s.v. or XML file. In the absence of SEC data files, users can instead enter values for 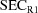 and 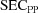. In place of DLS data files, users can enter radius, polydispersity, percent intensity and percent mass for the main peak. For gel-based experiments such as yield, SDS-PAGE purity and Lp, visual scores are required whether or not image files are loaded.

### 2.3. Results returned by the server

**Figure 3:**
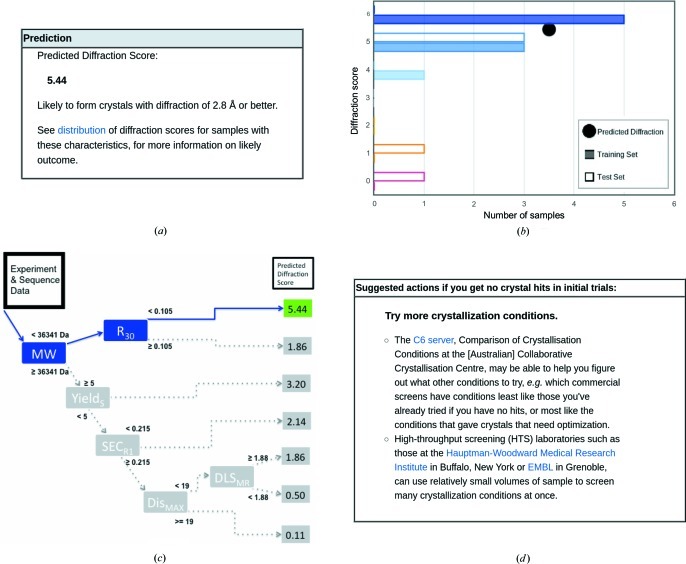
Predicted outcome and suggestions. (*a*) Average and (*b*) distribution of outcomes for protein samples with similar properties. Diffraction score: (0) no crystals; (1) no diffraction; (2) worse than 10.00 Å; (3) 10.00–4.01 Å; (4) 4.00–2.81 Å; (5) 2.80–2.01 Å; (6) 2.00 Å or better. (*c*) Path through the decision tree by which this sample was categorized. (*d*) Suggestions for further work on difficult targets.

Once sequence and experimental results are uploaded and selected, the user can submit the data attached to a sample for prediction of crystallization outcome. *PROSPERO* then runs the current predictor to generate outcome scores (Fig. 3 ▶). The server returns its prediction in several forms. It reports the experimental and sequence values used to categorize the samples, highlighting the positive or negative influence of key values on the predicted outcome. Below this is the predicted outcome (Fig. 3 ▶ *a*), the distribution of outcomes among the proteins in the training and test sets (Fig. 3 ▶ *b*), and the path through the decision tree by which this sample was categorized (Fig. 3 ▶ *c*). On the basis of that decision tree, the server also provides suggestions for ways to shift the protein from one category to another and thereby possibly improve crystallization outcome (Fig. 3 ▶ *d*).

#### 2.3.1. Mean outcome

The predicted outcome is based on the mean diffraction score for all samples in our training set with similar characteristics, *i.e.* those that follow the same path through the decision tree (Fig. 3 ▶ *c*). *PROSPERO* reports both the mean diffraction score and the interpretation, ranging from ‘not likely to form crystals’ to ‘likely to form crystals with diffraction of 2.8 Å or better’.

#### 2.3.2. Distribution of outcomes

Most categories of proteins are not uniform in their outcome, *e.g.* they contain a few samples that produced well diffracting crystals and many that did not. Therefore the actual distribution of outcomes for both our training (solid) and test (open) samples is shown, as in Fig. 3 ▶(*b*), so that users can judge the likelihood of attaining each level of success.

#### 2.3.3. Decision tree path

The key criteria that determine the path through the decision tree are highlighted (Fig. 3 ▶ *c*); these values are also shown with red or blue highlighting at the top of the prediction page, so you can see why your sample falls into its category.

#### 2.3.4. Suggestions

If the protein sample data fall into a category that is very likely to produce well diffracting crystals but no crystals have yet been obtained, *PROSPERO* provides links to potentially useful sites for determining other crystallization screens to try (Newman *et al.*, 2010 ▶) and to high-throughput screening facilities (Luft *et al.*, 2003 ▶; Mueller-Dieckmann, 2006 ▶; Dupeux *et al.*, 2011 ▶).

If the sample data fall into any other category, *PROSPERO* offers suggestions for changes in sequence or protocol which may help move the sample to a more successful category. Small changes can make large differences in crystallographic outcome (Derewenda, 2010 ▶). Removal of predicted disorder is an obvious example; less obvious suggestions include modifications to expression and purification protocols to improve the yield and/or the SEC profile. We have some evidence that changes such as those recommended by the server help in producing well diffracting crystals, but not enough cases to estimate how effective each change might be. Nevertheless, these suggestions are a good starting point for prioritizing further research when resources are limited.

## 3. *PROSPERO* as a tool for laboratory data handling

The data management and experimental analysis modules developed for *PROSPERO* may be useful laboratory tools in their own right. For example, the graphical representation of experimental characterizations shown in Fig. 2 ▶ illustrate standardized analysis and display of experimental data. The *PROSPERO* input modules allow measurements obtained using laboratory instruments with different manufacturer or model type to be imported into a standard format and then run through a standard set of curve-fitting or other processing procedures. This provides an alternative to idiosyncratic or proprietary processing and graphing routines provided with the individual instruments. Although only a simple data management infrastructure is currently implemented in *PROSPERO*, this framework may provide a useful open-source starting point for the development of a more sophisticated laboratory data management system.

## 4. Availability

The source and documentation for input modules used to analyze DSF and SEC curves are available from the ‘Download’ tab of the *PROSPERO* menu bar, as are sample input files and the templates, forms and scripts for the *Catalyst* web interface (http://www.catalystframework.org) and schema for the MySQL database for the site. The source code is currently available under the Artistic License, but other licensing arrangements are possible by request.
